# Comparative Analysis of Bacteriome in Hair Follicle Layers of Patients with Female Pattern Androgenic Alopecia

**DOI:** 10.3390/microorganisms13061365

**Published:** 2025-06-12

**Authors:** Yujun Park, Seoyeon Kyung, Seyoung Mun, Byung Sun Yu, Kyengeui Yun, Chaeyun Baek, Dong-Geol Lee, Seunghyun Kang, Soon Re Kim, Ju-Hee Kim, Yeji Lee, Byung-Cheol Park, Kyudong Han

**Affiliations:** 1 Department of Microbiology, College of Science & Technology, Dankook University, Cheonan 31116, Republic of Korea; qkrwodbwns1109@gmail.com (Y.P.); ybs901287@gmail.com (B.S.Y.); sindy@hunbiome.com (K.Y.); leedg@cosmax.com (D.-G.L.); heyji4016@naver.com (Y.L.); 2 R&I Center, COSMAX BTI, Pangyo-ro 255, Bundang-gu, Seongnam 13486, Republic of Korea; sykyung@cosmax.com (S.K.); cybaek@cosmax.com (C.B.); shyunk@cosmax.com (S.K.); 3 Department of Cosmedical & Materials, Dankook University, Cheonan 31116, Republic of Korea; munseyoung@gmail.com; 4 Center for Bio Medical Engineering Core Facility, Dankook University, Cheonan 31116, Republic of Korea; 5 Smart Animal Bio Institute, Dankook University, Cheonan 31116, Republic of Korea; 6 Department of Human Microbiome Research HuNbiome Co., Ltd., R&D Center, Seoul 08504, Republic of Korea; 7 Department of Dermatology, College of Medicine, Dankook University, Cheonan 31116, Republic of Korea; sl715@nate.com (S.R.K.); wn3355@naver.com (J.-H.K.); 8 Dermato-Translational Research Institute, Dankook University, Cheonan 31116, Republic of Korea

**Keywords:** alopecia, androgenetic alopecia, female pattern hair loss, male pattern hair loss, microbiome analysis, hair follicle layer microbiome

## Abstract

Androgenetic alopecia (AGA) is the most common form of patterned hair loss, exhibiting gender-specific clinical features. Recent studies highlight the importance of the skin microbiome in maintaining skin health, but the relationship between the hair follicle microbiome and hair loss, particularly AGA, remains understudied. Hair follicle layer samples were collected directly from the crown region of female pattern hair loss (FPHL), male pattern hair loss (MPHL), and healthy adult women (control) groups. Microbial DNA was extracted and analyzed using Illumina 16S rRNA V3–V4 gene amplicon sequencing. Alpha-diversity and beta-diversity analyses and taxonomic and functional profiling were conducted through relative abundance, LEfSe, and PICRUSt2 analyses. The alpha-diversity analysis showed a significant decrease in microbial richness in the hair loss groups. Unweighted UniFrac-based beta-diversity analysis revealed significant clustering between the control group and the FPHL group. Taxonomic profiling and LEfSe analysis identified differences in microbial composition and biomarkers. PICRUSt2 analysis further revealed altered pathways related to porphyrin metabolism, fatty acid biosynthesis, and steroid hormone metabolism. Additionally, differences in microbiome composition and potential functions were found between the FPHL and MPHL groups. This study provides comprehensive insights into the hair follicle microbiome, revealing unique microbial patterns and functional alterations associated with FPHL. Understanding these microbiome characteristics may contribute to targeted approaches for addressing AGA. Further research is warranted.

## 1. Introduction

Alopecia, including androgenetic alopecia (AGA), alopecia areata (AA), cicatricial alopecia, and telogen effluvium, is a significant concern in modern society, classified by clinical pattern, site, and underlying cause [1,2,3,4]. Alopecia results from complex interactions between internal factors—such as genetic, physiological, immune, hormonal, autoimmune, and psychological factors—and external environmental influences [5]. AGA is the most common form of patterned alopecia, characterized by the gradual miniaturization of hair follicles after the growth phase, influenced by steroid sex hormones, family genetic history, and external factors [6,7]. AGA presents differently in males and females: in females as female pattern hair loss (FPHL), typically affecting the vertex, and in males as male pattern hair loss (MPHL), with M-shaped recession at the forehead and vertex [8]. Furthermore, MPHL displays a more distinct pattern, primarily influenced by steroid hormones and genetic factors, while FPHL demonstrates a poorer response to pharmacological treatments and has fewer available therapeutic options [9]. Despite its high prevalence, FPHL remains under-investigated at the molecular and microbial levels, particularly regarding the follicular microenvironment [10].

The scalp, as a specialized skin site with high follicular density and sebaceous activity, harbors distinct microbial communities that dynamically respond to environmental factors such as pH, temperature, moisture, and sebum production [11]. Accordingly, recent studies have increasingly focused on the scalp microbiome in order to understand its potential role in hair-related disorders. However, most of these studies have relied on surface-level sampling methods such as swabs, which may not fully capture the microbial communities residing within the follicular compartment.

Both commensal and opportunistic microbes can modulate local immune activity, particularly within skin-associated structures [12]. Yet, the extent to which follicle-residing microorganisms influence perifollicular immune responses or contribute to inflammatory dysregulation in alopecia remains unclear. Despite the high prevalence of FPHL, most microbiome studies have focused on male AGA populations, leaving a significant gap in our understanding of female-specific follicular microbial patterns [10].

Recent studies on the microbiome have demonstrated that alterations in microbial composition and diversity are associated with the development of skin conditions such as acne, atopic dermatitis, psoriasis, and dandruff [13,14]. A study involving Korean AGA patients proposed that various external factors modulate the relationship between the scalp microbiome and AGA, the nature of which remains to be fully elucidated [15]. In Japanese male patients with AGA, the scalp microbiome exhibited an increased abundance of *Cutibacterium* and *Malassezia restricta*, a fungus capable of consuming palmitic acid, and a decreased abundance of *Corynebacterium* [16]. Previous studies comparing normal and miniaturized hair follicles in Singaporean Asian male AGA patients reported a higher abundance of *Cutibacterium acnes* in miniaturized follicles, accompanied by the upregulated expression of immune response genes [17]. Although direct causality remains unclear, microbial imbalances within the follicular niche may be associated with altered immune signaling and hair growth regulation [18].

Hair growth is sustained by the continuous division, proliferation, and differentiation of germinal matrix cells, which closely interact with dermal papilla cells located at the base of the hair bulb [19,20]. The hair shaft forms through keratinization and remains anchored in the follicle [21,22,23]. These cells exchange nutrients and signals with dermal papillae via surrounding capillaries and nerve endings [24,25]. In contrast to the follicular microenvironment, the scalp surface is drier, more acidic, and more exposed to UV radiation. The dry environment of the scalp is less supportive of microbial survival, thereby reducing microbial diversity. In contrast, hair follicles create a relatively anaerobic microenvironment that favors the proliferation of anaerobic microorganisms [26,27,28]. However, most existing studies have focused predominantly on the scalp-level microbiome.

The follicular niche interfaces directly with the dermal papilla, germinal matrix, and immune cells, underscoring its functional distinctiveness [29]. These interactions are central to hair cycle regulation, growth factor signaling, and local immune responses. Given these intimate anatomical and functional interactions, microbial activity within the follicular niche is more likely to exert a direct influence on hair follicle physiology and pathology than that of surface-residing microbes [30,31]. In light of the symbiotic relationship between the follicular microbiome and host cells, including dermal papillae and germinal matrix cells, this study specifically focused on characterizing the microbiome within the hair follicle layer. In this context, the “hair follicle layer” refers to the segment of the hair shaft that remains embedded within the scalp tissue at the time of plucking, including structures in direct contact with the follicular epithelium. To minimize environmental contamination, only the embedded portion of the hair shaft obtained by direct plucking was retained for downstream analysis.

Given the insufficient understanding of microbial alterations in FPHL, this study primarily aimed to characterize the bacterial communities residing in the hair follicle layer of FPHL patients and healthy women. To distinguish disease-specific microbial features in women, we compared the hair follicle microbiome between FPHL patients and healthy adult women. Additionally, an MPHL group was included to investigate whether sex-specific differences in AGA pathophysiology were also reflected in the follicular microbiome, despite the absence of a male control group.

## 2. Materials and Methods

### 2.1. Clinical Patient Demographics and Hair Follicle Layer Sample Collection

Hair follicle layer samples were collected through the Department of Dermatology Research Institute at the Dankook University Hospital, College of Medicine. The study included adult men and women aged between 18 and 65 diagnosed with androgenic alopecia, defined by the BASP classification with at least the M1, C1, or U1 stages, and V1 or F1 for specific types [32]. For male patients, the diagnosis was based on a Norwood–Hamilton classification of at least stage 2 or 2A, while for female patients, a Ludwig classification of at least stage 1 was required [33,34,35]. Additionally, healthy adult women between 18 and 65 years of age with no clinical diagnosis of alopecia or scalp disorders were recruited as the control group, in accordance with the IRB-approved protocol.

The MPHL group was included to examine potential sex-based differences in the follicular microbiome of AGA patients. Although a male control group was not recruited, comparative analysis across FPHL patients, MPHL patients, and healthy female participants enabled us to distinguish disease-specific microbial features in women and explore male–female differences in the context of the disease.

Hair follicle layer samples were collected from the FPHL group (n = 30), MPHL group (n = 20), and control group (n = 20). The average ages of the participants were 46.3 years for the FPHL group, 45.2 years for the MPHL group, and 42.7 years for the control group (Supplementary Table S1). The specific clinical stage per patient was not recorded; inclusion was based on meeting the minimum diagnostic criteria.

### 2.2. Participant Exclusion Criteria and Sample Processing

Hair follicle layer samples were collected specifically from the crown region of the scalp. From each participant, three hairs were plucked at each of five distinct sites at least 5 cm apart, totaling approximately 10–15 hairs. All the samples were collected using a standardized pluck method without ethanol treatment. Participants were instructed to refrain from shampooing for 24 h before sample collection. The plucked hair follicle layer samples were placed in 1.5 mL tubes containing CD1 buffer from the QIAGEN PowerFecal Pro DNA Kit (QIAGEN, Hilden, Germany) and stored at −80 °C in CD1 buffer until microbial DNA was extracted. This study received approval from the Dankook University Hospital Institutional Review Board (IRB protocol number: DM07C042022, approved on 28 April 2022) in accordance with academic research standards. All clinical experiments conducted in this study were performed in accordance with the guidelines and regulations outlined in the Declaration of Helsinki [36].

Participants with the following conditions were excluded from the analysis: (1) psychiatric disorders; (2) topical corticosteroid preparations applied to the scalp within one month prior to sample collection; (3) topical hair loss treatments or hair growth stimulants used within one month; (4) the use of Dutasteride or Finasteride within the previous three months; (5) severe seborrheic dermatitis, scalp psoriasis, or scalp infections; (6) individuals with other hair disorders, such as telogen effluvium or alopecia areata, were excluded based on clinical evaluation by a board-certified dermatologist. Differential diagnosis was confirmed using patients’ medical histories, clinical pattern assessment, and trichoscopic findings, in accordance with the established diagnostic criteria for androgenic alopecia. 

### 2.3. Bacterial Genomic DNA Extraction and Quality Control

Microbial DNA was isolated from hair follicle layer samples using the QIAGEN PowerFecal Pro DNA Kit (QIAGEN, Hilden, Germany; Cat. No. 51804). All experimental procedures were performed according to the recommended protocols provided with the QIAGEN PowerFecal Pro DNA Kit. The quality of the isolated microbial DNA was assessed using a Qubit fluorometer (Qubit Flex, Invitrogen, Carlsbad, CA, USA). Samples were stored at −80 °C prior to library preparation.

### 2.4. Illumina 16S Ribosomal RNA Gene V3–V4 Amplicon Sequencing Library Preparation and Sequencing

Sequencing of the 16S rRNA gene was conducted following the official Illumina protocol (Illumina, San Diego, CA, USA). Library construction for Illumina 16S rRNA gene metagenomic sequencing targeted the V3–V4 hypervariable region. Metagenomic DNA amplification targeting the V3–V4 region was performed using KAPA Hot Start Ready Mix (2X) (Roche, Basel, Switzerland), following Illumina’s recommended procedures. The primer sequences used for sequencing library construction were as follows: 16S rRNA gene 314F primer: 5′-TCGTCGGCAGCGTCAGATGTGTATAAGAGACAGCCTACGGGNGGCWGCAG-3′; 16S rRNA gene 806R primer: 5′-GTCTCGTGGGCTCGGAGATGTGTATAAGAGACAGGACTACHVGGGTATCTAATCC-3′ [37]. PCR amplification of the 16S rRNA gene V3–V4 region was performed using a thermal cycler under the following conditions: initial denaturation at 95 °C for 5 min, followed by 38 cycles of denaturation at 95 °C for 30 s, annealing at 55 °C for 40 s, and extension at 72 °C for 50 s. A final extension step was carried out at 72 °C for 3 min, and the reaction was then held at 4 °C. The number of amplification cycles (38) was optimized for skin-derived samples, reflecting the lower microbial biomass typically present in these specimens. After PCR amplification, PCR products were purified with AMPure XP beads (Beckman Coulter, Pasadena, CA, USA). Library construction was performed using the Nextera XT Index Kit (Illumina, San Diego, CA, USA), and the resulting libraries were purified using AMPure XP beads (Beckman Coulter, Brea, CA, USA). The metagenomic sequencing of each library was performed using the paired-end 2 × 300 bp Illumina MiSeq™ protocol.

### 2.5. 16S rRNA Gene V3–V4 ASV Data Processing Workflow

The microbiome sequencing data obtained using the Illumina MiSeq™ platform (Illumina, San Diego, CA, USA) were processed through the QIIME2 pipeline (version 2022.8) [38]. Prior to applying the DADA2 (Divisive Amplicon Denoising Algorithm 2, version 1.22.0) plugin, optimal trimming parameters based on sequence quality profiles were determined using the FIGARO tool (https://github.com/Zymo-Research/figaro, accessed on 10 March 2023) [39]. Denoising and amplicon sequence variant (ASV) inference were then performed using the DADA2 plugin with the following parameters: forward and reverse reads were truncated at 240 bp and 200 bp, respectively; the first 17 bases of the forward reads and 21 bases of the reverse reads were trimmed; reads with more than 2 expected errors or with a quality score ≤ 2 were removed; and a minimum overlap of 12 nucleotides was required for read merging. Chimeric sequences were identified and removed using the “consensus” method implemented in DADA2, resulting in high-confidence ASVs.

The taxonomic classification of the ASVs was conducted using a Naïve Bayes classifier trained on the 99% non-redundant SILVA 138v rRNA reference database (released on 16 December 2019), precisely trimmed to match the 16S gene rRNA V3–V4 hypervariable region, following the experimental design in [40]. Features assigned to Archaea, Eukaryota, Mitochondria, or Chloroplasts were excluded. The remaining bacterial ASVs were used for downstream phylogenetic tree construction (via the align-to-tree-mafft-fasttree plugin) and compositional taxonomic analysis. Following taxonomic filtering, the ASV tables were rarefied to 2574 reads per sample to normalize the sequencing depth for downstream diversity and compositional analyses. This value corresponded to the lowest sequencing depth among all the samples and was selected in order to retain the full dataset without introducing bias from unequal read depths. Although rarefaction can reduce statistical power, our chosen threshold exceeded the minimum depth (1000 reads) previously recommended as sufficient for reliable microbial diversity estimation [41].

### 2.6. Evaluation of Alpha-Diversity and Beta-Diversity in FPHL, MPHL, and Control Groups

Alpha-diversity indices—including observed features (representing observed ASV richness); the Chao1 index, which estimates theoretical species richness by accounting for undetected rare taxa; the Shannon index, for assessing overall diversity (considering both richness and evenness); the Simpson index (original form, D); and Pielou’s evenness for evaluating community evenness—were used to characterize microbial community structure in the FPHL, MPHL, and control groups. The Simpson index (D), defined as the probability that two individuals randomly selected from a sample belong to the same species, was interpreted such that lower values indicated higher diversity or evenness. Statistical comparisons between groups were conducted using the Kruskal–Wallis test followed by Mann–Whitney U tests for the alpha-diversity metrics [42,43,44,45]. Beta-diversity dissimilarities between the groups were assessed using unweighted UniFrac distances, which account for phylogenetic relationships without considering abundance, and Bray–Curtis distances, which reflect differences based on relative microbial abundance. Principal Coordinate Analysis (PCoA) was used to visualize dissimilarity patterns between samples. Group-level differences in beta-diversity were evaluated using permutational multivariate analysis of variance (PERMANOVA, 999 permutations), as implemented in the R packages vegan and adonis [46,47,48,49]. All the alpha- and beta-diversity analyses were performed at the ASV level using rarefied feature tables.

### 2.7. Bacterial Relative Abundance in FPHL, MPHL, and Control Groups

Bacterial relative abundance was assessed using the classification table generated from the ASV-level taxonomic assignments filtered based on confidence scores. Relative abundances at each taxonomic rank were calculated as the proportion of each taxon within the individual samples. This approach enabled the identification of compositional differences among the FPHL, MPHL, and control groups.

All the taxonomic annotations reflect the nomenclature used in the SILVA 138v database. Recent updates to the SILVA taxonomy (e.g., the division of Firmicutes into Firmicutes_A and Firmicutes_B) were not applied in this study in order to maintain consistency with the original analysis pipeline.

### 2.8. Taxonomic and Functional Profiling of Microbiome Using LEfSe and PICRUSt2 Analysis

Linear discriminant analysis (LDA) effect size (LEfSe) analysis was conducted in order to identify bacterial features that were significantly enriched in the FPHL, MPHL, and control groups [50]. LEfSe tool was deployed using the command-line version provided via the bioconda channel, with default parameters (sum normalization = 1,000,000; alpha = 0.05 for the Kruskal–Wallis test; LDA score threshold = 2.0). Taxonomic features across multiple levels (from phylum to species) were included in the analysis, and significant features were identified based on both statistical significance (*p* < 0.05) and biological relevance (LDA score ≥ 2.0). While discriminative features were identified across the full taxonomic spectrum (from phylum to species), genus- and species-level taxa were primarily evaluated in downstream analyses, as they exhibited the most prominent group-specific differences.

Based on the ASV taxonomy and count table, PICRUSt2 (v2.5.2) [51] was used to predict the potential functional profiles of the microbiomes. Functional prediction was performed using the default PICRUSt2-oldIMG reference database, which is based on KEGG Orthology (KO) annotations. Differential abundance analysis of the predicted KEGG pathways was conducted using the Kruskal–Wallis test implemented in the ALDEx2 framework via the ggpicrust R package (R version 4.1.3) [52,53]. *p*-values were adjusted for multiple testing using the Benjamini–Hochberg (BH) method, and significantly different pathways were identified based on an adjusted *p*-value (*q*-value) threshold of ≤0.05.

## 3. Results

### 3.1. 16S rRNA Gene V3–V4 Sequencing Data Processing of Clinical Samples

Microbial gDNA samples were used to prepare Illumina sequencing libraries targeting the 16S rRNA gene V3–V4 hypervariable region. Next-generation sequencing (NGS) generated 300 bp paired-end short-read amplicon sequencing data. The sequencing results yielded an average of 35,311 demultiplexed reads per sample. After applying the DADA2 pipeline, the average denoised read count per sample was 35,035. The average number of pre-processed reads with a quality score of Q30 or higher (average non-chimeric reads) was 30,290 (Supplementary Table S1). Based on the pre-processed reads, classification using the SILVA 138v 16S rRNA gene reference database resulted in 572 bacterial ASVs at the species level and 319 at the genus level (sequence alignment above the 70% confidence threshold; Supplementary Table S2).

### 3.2. Alpha-Diversity and Beta-Diversity of Hair Follicle Layer Microbiome

**Figure 1:**
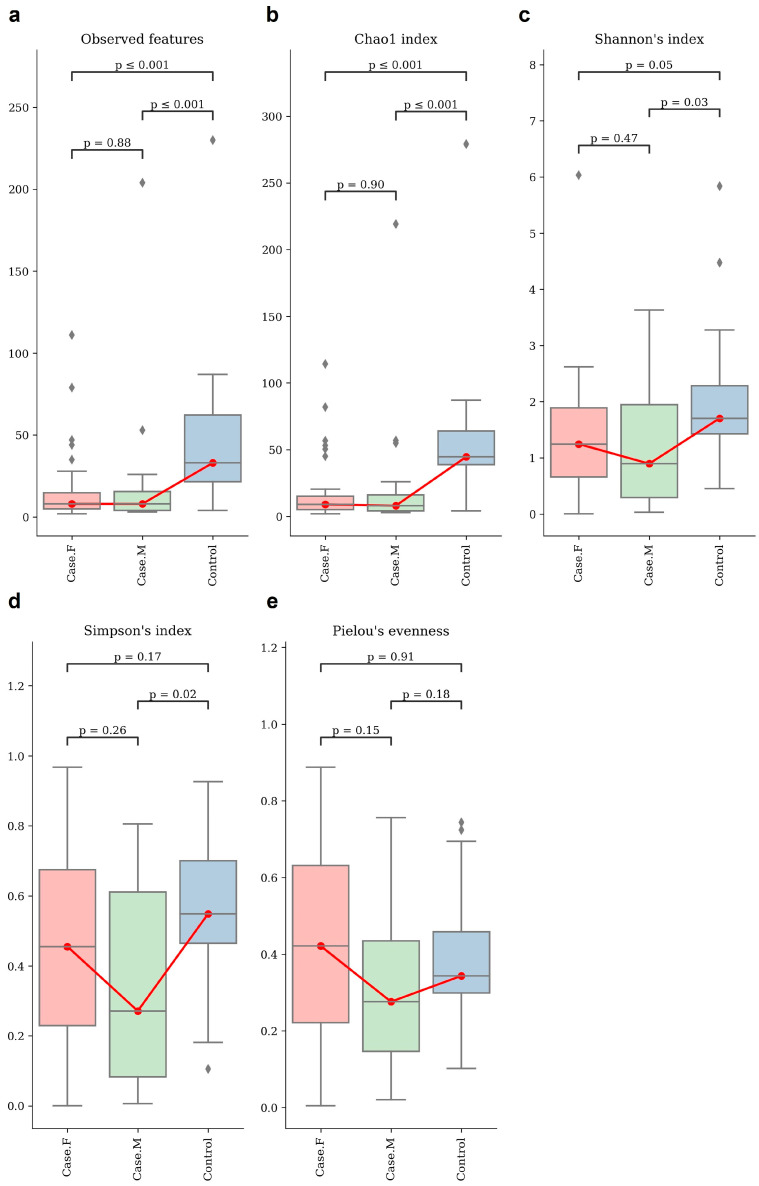
The alpha–diversity analysis of the hair follicle layer in each group. These box plots show the alpha–diversity estimation scores of each group, which were calculated using (**a**) observed features, which represent the number of unique ASVs detected per sample and serve as a proxy for observed species richness; (**b**) Chao1 index; (**c**) Shannon’s index; (**d**) Simpson’s index; and (**e**) Pielou’s evenness. Group comparisons were evaluated using statistical analysis (Kruskal–Wallis, Mann–Whitney). For some indices, no significant differences were observed between the individual groups. However, there appears to be distinct diversity between healthy individuals and hair loss patients. Red box plot: Case.F, FPHL group. Green box plot: Case.M, MPHL group. Blue box plot: Control group. The red lines connect the mean values of each group in order to indicate directional trends across the groups. Diamond symbols indicate outliers beyond the interquartile range.

Alpha-diversity was assessed using the observed features and Chao1, Shannon’s, Simpson’s, and Pielou’s evenness indices (Figure 1; Supplementary Table S3). Statistical significance was evaluated using Mann–Whitney U tests (Supplementary Table S4). The control group showed a significantly higher richness than both the FPHL and MPHL groups, as indicated by the observed features and the Chao1 index. The FPHL group had the lowest richness, though the difference between the FPHL and MPHL groups was not statistically significant.

For diversity, the Shannon index revealed the highest diversity to be in the control group and the lowest to be in the MPHL group. Significant differences were found between the control group and both hair loss groups, but not between FPHL and MPHL. In terms of evenness, the Simpson index differed significantly between the control and MPHL groups, while Pielou’s evenness showed no significant differences among the groups.

**Figure 2:**
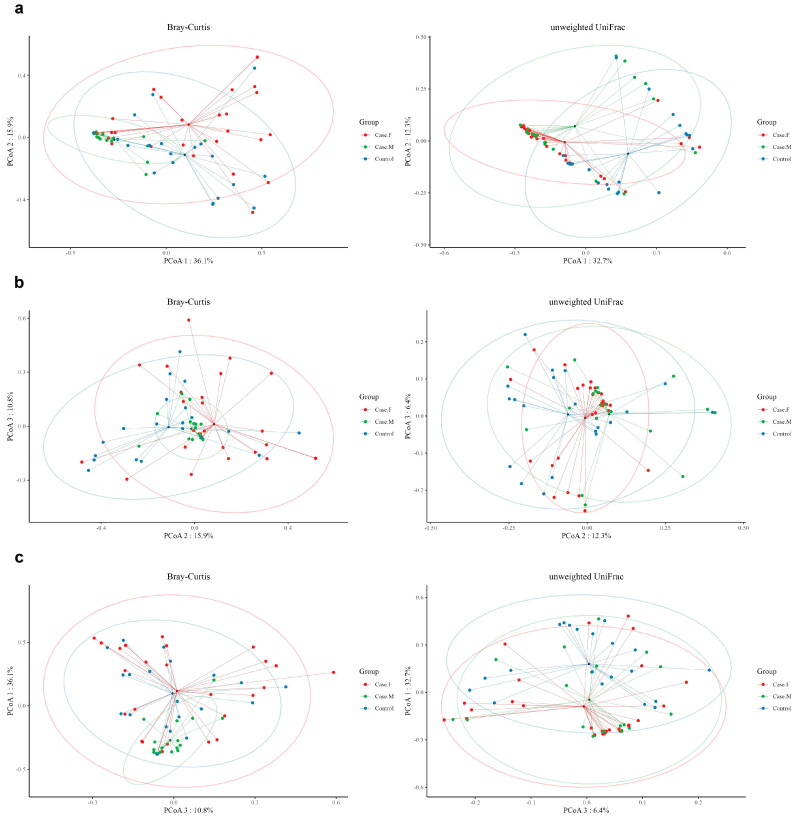
The beta-diversity analysis of the hair follicle layer between groups. The microbial beta-diversity analysis measured by both Bray–Curtis distance and the unweighted UniFrac distance matrix for all groups (Case.F: FPHL group; Case.M: MPHL group; control group). The unweighted UniFrac distance analysis showed significant *p*-values of 0.001 for comparisons between Case.F vs. Case.M vs. control, control vs. Case.M, and control vs. Case.F. However, the Case.M vs. Case.F comparison was not significant. In contrast, using Bray–Curtis distance, significant results (*p*-value = 0.001) were observed for Case.F vs. Case.M vs. control, control vs. Case.M, and Case.M vs. Case.F, while no significant differences were found between the control and female groups. (**a**): Comparison between PCoA 1 and PCoA 2; (**b**): comparison between PCoA 2 and PCoA 3. (**c**): Comparison between PCoA 1 and PCoA 3. Red dot: Case.F, FPHL group. Green dot: Case.M, MPHL group. Blue dot: Control group.

Beta-diversity was examined using unweighted UniFrac and Bray–Curtis distances (Figure 2; Supplementary Table S5). Bray–Curtis analysis indicated significant differences in microbial composition between the MPHL group and both the FPHL and control groups. In contrast, the unweighted UniFrac distances revealed significant differences between the control group and each of the FPHL and MPHL groups, but not between the FPHL and MPHL groups.

### 3.3. Relative Abundance in Hair Follicle Layer Microbiome

**Figure 3:**
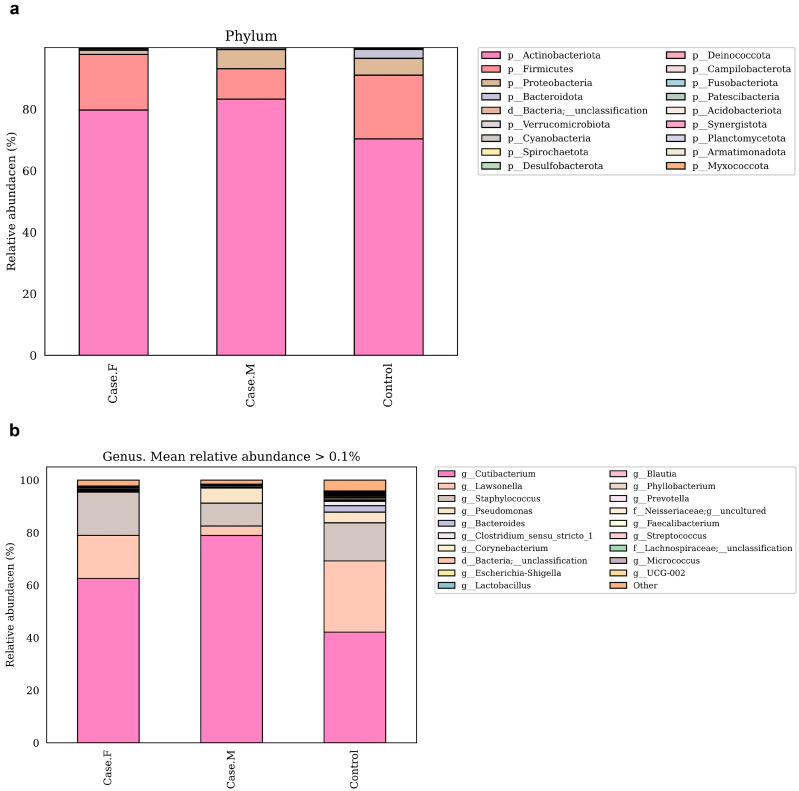
These bar plots show the relative abundance of bacterial taxa in the hair follicle layer microbiota at the phylum (**a**) and genus (**b**) levels, based on 16S rRNA V3–V4 sequencing. The taxa shown in the legend are ordered by relative abundance within each group. Taxonomic annotations from phylum to species were assigned using the SILVA 138v reference classifier. Some taxon names (e.g., Firmicutes) may correspond to updated nomenclature (e.g., Firmicutes_A, Firmicutes_B) in later SILVA releases, and similar changes may apply at the genus and species levels. Case.F: FPHL group; Case.M: MPHL group; Control: healthy.

To compare microbial composition between groups, taxonomic analysis was performed at the phylum and genus levels (Figure 3a,b; Supplementary Table S6). Species-level analysis has been excluded from the main text due to the limited taxonomic resolution of the 16S rRNA V3–V4 region. At the phylum level, Actinobacteriota, Firmicutes, and Proteobacteria were the most abundant. Actinobacteriota was enriched in the FPHL (79.6%) and MPHL (83.2%) groups compared to the control group (70.2%). Firmicutes was decreased in the MPHL group (9.8%) relative to the control (20.7%) and FPHL (18%) groups. In contrast, Proteobacteria was lower in the FPHL group (1.3%) than in the control (5.4%) and MPHL (6.2%) groups.

At the genus level, the dominant taxa were *Cutibacterium*, *Lawsonella*, *Staphylococcus*, and *Pseudomonas*. These findings were further contextualized by reviewing previously reported abundance trends in other scalp diseases, as is discussed later in the manuscript. *Cutibacterium* was most abundant in the MPHL group (79%), followed by the FPHL (62.6%) and control (42.1%) groups. This pattern aligns with previous studies on MPHL [16], although some studies have reported reductions in both FPHL and MPHL groups [15]. Additionally, *Cutibacterium* has been reported to decrease in other scalp conditions [54,55,56]. *Lawsonella* showed reduced abundance in both the FPHL (16.4%) and MPHL (3.5%) groups relative to the control group (27.1%), consistent with findings in AGA and atopic scalp dermatitis [15,55]. The MPHL group exhibited particularly low levels. No significant reduction in *Staphylococcus* was observed between the FPHL and control groups, in contrast to previous reports on alopecia areata and AGA [15,57,58]. *Pseudomonas* was markedly reduced in the FPHL group (0.1%) compared to the control (4%) and MPHL (5.8%) groups. This pattern is consistent with previous findings specific to FPHL [15]. *Corynebacterium* was less abundant in the FPHL group (0.14%) compared to the MPHL (0.5%) and control (0.25%) groups, supporting prior studies [15,16]. *Micrococcus* also showed decreased relative abundance in both the FPHL (0.07%) and MPHL (0.01%) groups compared to the control group (0.26%), consistent with previous reports on female scalps [15].

Together, these six genera accounted for 95.8% of the total abundance in the FPHL group, 97.5% in the MPHL group, and 88.3% in the control group.

### 3.4. Significant Microbiota Biomarkers Identified Using LEfSe Analysis

**Figure 4:**
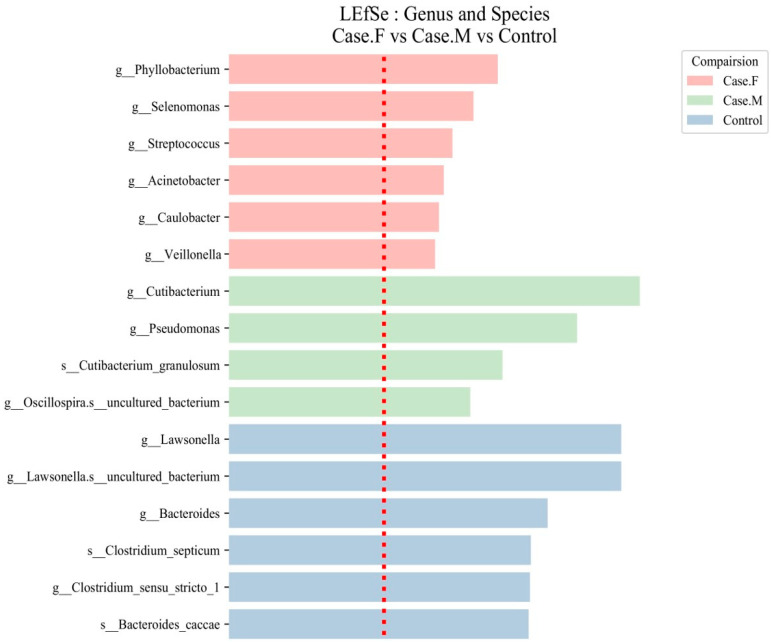
LEfSe analysis of the hair follicle layer microbiome in patients with AGA compared to healthy controls. LEfSe identified bacterial clades that were differentially abundant among the FPHL group (Case.F), the MPHL group (Case.M), and the control group. The clades shown in the graph met both a statistical significance threshold (*p* < 0.05) and a linear discriminant analysis (LDA) score threshold of >2, indicating their meaningful effect size. The taxonomic labels are prefixed with *g__* for genus and *s__* for species. The red dotted line represents the LDA score threshold of 2.0.

Using the LEfSe tool, distinctive microbial features were identified in the FPHL, MPHL, and control groups using an LDA score threshold of ≥2 (Figure 4; Table 1; Supplementary Table S7). At the genus and species levels, 6 groups were identified in the FPHL group, 4 in the MPHL group, and 41 in the control group, totaling 51 groups.

In the FPHL group, six genera were identified: *Phyllobacterium*, *Selenomonas*, *Streptococcus*, *Acinetobacter*, *Caulobacter*, and *Veillonella*. In the MPHL group, three genera were identified: *Cutibacterium*, *Pseudomonas*, and *Oscillospira*. Additionally, *Cutibacterium granulosum* was detected at the species level. In the control group, taxa such as *Lawsonella*, *Bacteroides*, *Clostridium sensu stricto 1*, and *Bacteroides caccae* were among the most discriminative features.

Although a total of 41 discriminative taxa were identified in the control group, only the top 6 taxa with the highest LDA scores are included in Table 1 for clarity, as is also indicated in the table caption. The full list of taxa is provided in Supplementary Table S7. Species-level taxa, including those identified in both the MPHL (e.g., *Cutibacterium granulosum*) and control groups (e.g., *Bacteroides caccae*, *Clostridium septicum*), should be interpreted with caution due to the limited taxonomic resolution of the 16S rRNA V3–V4 region.

### 3.5. In Silico Functional Analysis Using PICRUSt2

**Figure 5:**
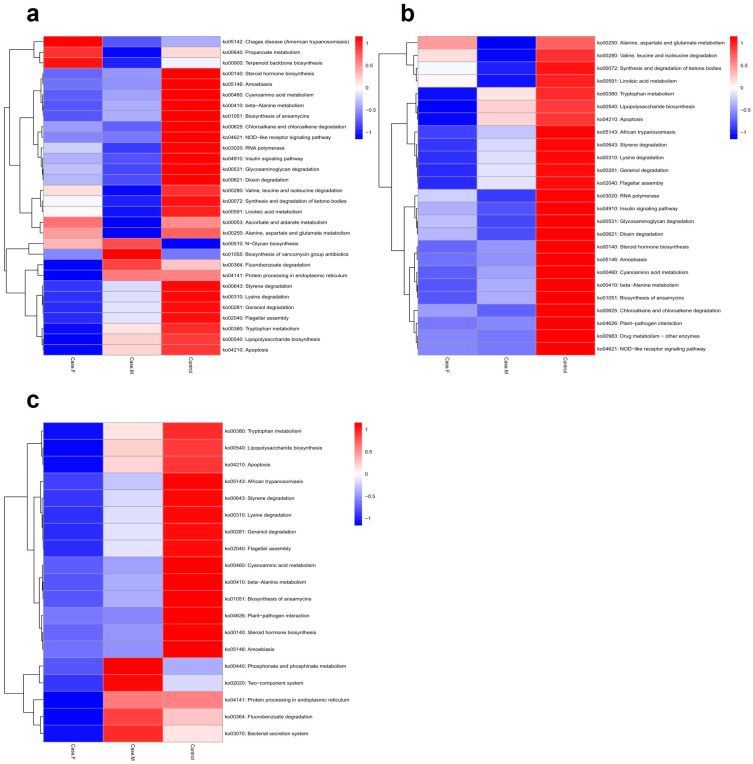
The functional analysis results, represented by a heatmap. The predicted gene functions of the bacteria were analyzed through the PICRUSt2 algorithm. (**a**): A heatmap for the top 30 KEGG Orthology results determined by *p*–values. (**b**): A heatmap for the list of bacteria showing lower abundance in the AGA groups compared to control. (**c**): A heatmap for the list of bacteria showing lower abundance in the FPHL group compared to the other groups.

To predict potential microbiome functions in the FPHL, MPHL, and control groups, PICRUSt2 was utilized to identify 102 significant KEGG pathways (*p*-value ≤ 0.05) (Figure 5; Supplementary Table S8). Using a *p*-value threshold of ≤0.05, the FPHL group exhibited higher values than the MPHL group in 35 pathways and higher values than the control group in 72 pathways. Among these, 24 pathways exhibited higher values than both the MPHL and control groups. The FPHL group exhibited lower values than the MPHL group in 67 pathways and lower values than the control group in 30 pathways, with 19 pathways showing lower values than in both of the other groups (Supplementary Table S8).

## 4. Discussion

Our analysis of the hair follicle layer microbiome revealed distinct alpha-diversity patterns compared to previous scalp-level studies, providing new insights into the localized microbial ecology of hair follicles in AGA. While earlier reports noted increased richness in FPHL scalp microbiota, our findings showed reduced richness in both FPHL and MPHL follicular microbiomes, contrasting with the previous scalp microbiome results reported by Jung et al. [15]. This discrepancy suggests anatomical variation in microbial colonization, potentially reflecting unique follicle-specific ecological dynamics. Although the differences in evenness were less pronounced, a tendency toward lower community evenness in the FPHL group was observed, which contrasts with previous scalp microbiome studies that report higher evenness in FPHL patients [15]. Based on these findings, we hypothesize that the microbial ecology of the follicle layer in FPHL may differ from that of the scalp. Since a direct comparison between MPHL patients and healthy adult males was not available in this study, it remains uncertain whether a similar divergence would be observed in male follicular microbiota; however, such a possibility cannot be excluded.

In our findings, *Cutibacterium*, *Lawsonella*, *Staphylococcus*, and *Pseudomonas* were the most abundant genera, consistent with previous studies identifying *Cutibacterium* and *Staphylococcus* as prevalent genera on the scalp [59,60]. *Cutibacterium* was particularly abundant in the MPHL group, which aligns with previous studies [16]. However, contrasting findings have reported decreased levels of *Cutibacterium* in both FPHL and MPHL groups [15], as well as in other scalp conditions such as seborrheic dermatitis and dandruff [54,55,56]. *Cutibacterium*, primarily located within hair follicles, is a major component of the follicular microbiome. It contributes to skin health by regulating lipid metabolism, modulating immunity, and reducing oxidative stress [61,62,63]. However, it is also implicated in dermatologic conditions such as acne vulgaris, AGA, dandruff, and seborrheic dermatitis [17,57,64,65,66]. This dual role of *Cutibacterium* underscores the complex ecological balance of the follicular niche, where microbial communities dynamically respond to local immune and environmental cues.

To provide context for our findings, a literature-based summary of genus-level trends observed in various scalp-related conditions is presented in Table 2. This table highlights how *Cutibacterium* and *Staphylococcus*, among others, exhibit variable abundance patterns beyond AGA, such as in seborrheic dermatitis, dandruff, and alopecia areata. In particular, *Staphylococcus* has often been found to be reduced in hair loss patients, showing differential trends across multiple scalp diseases.

*Lawsonella* was reduced in both the FPHL and MPHL groups. This pattern is in agreement with prior studies showing reduced *Lawsonella* in AGA and atopic scalp dermatitis [15,55]. *Staphylococcus* was not significantly different between the FPHL and control groups. This finding contrasts with earlier studies reporting decreased *Staphylococcus* in AA and AGA [15,57,58]. *Pseudomonas* was substantially reduced in the FPHL group but not in the MPHL group. This suggests that *Pseudomonas* may serve as a potential microbial biomarker distinguishing FPHL from MPHL [15].

*Corynebacterium* was less abundant in the FPHL group. *Micrococcus* levels were also lower in both hair loss groups compared to controls, supporting previous observations of female scalps [15]. Further research is needed to confirm the association between hair loss and the composition of *Staphylococcus*, including well-known skin-associated species such as *Staphylococcus aureus*, *Staphylococcus capitis*, and *Staphylococcus epidermidis* [66,68,69,70,71].

Probiotics are important for the treatment and prevention of gastrointestinal disorders and can exert effects beyond the gut, influencing overall health and skin function [72,73]. *Lactobacillus amylovorus*, which is known to produce antifungal substances, was nearly absent in the FPHL (0%) and MPHL (0.04%) groups, compared to a presence of 0.24% in the control group [74]. These findings raise the possibility that probiotic-derived bioactive substances could help to suppress fungal colonization (e.g., *Malassezia*) and support beneficial microbiota within the follicular environment. *Micrococcus luteus*, another commensal known to produce skin-beneficial substances, was also reduced in both the FPHL and MPHL groups, warranting further investigation into its functional roles in skin homeostasis and the potential implications of its loss in hair loss conditions [75].

LEfSe analysis revealed that *Phyllobacterium*, found exclusively in the FPHL group, is known to disrupt the skin microbiota cluster in rats and is recognized for its secretion of lipopolysaccharides (LPSs) [76,77]. LPSs can promote the production of pro-inflammatory cytokines such as IL-6, TNF-α, and nitric oxide, while inhibiting anti-inflammatory cytokines such as IL-10 [78,79]. Additionally, LPSs can activate the NF-κB pathway by upregulating the NOD2 gene, thereby regulating inflammation and immune responses [80]. Considering these aspects and the relative abundance data in Table 1, the exclusive presence of *Phyllobacterium* in the FPHL group suggests its potential role as a causative microorganism contributing to microbial imbalances or distinct patterns of AGA in FPHL as compared to MPHL.

Through PICRUSt2 analysis, several microbial pathways associated with AGA were identified. The presence of porphyrin, produced by *C. acnes* within pilosebaceous units, may be elevated in the AGA group due to increased activity within the porphyrin-related components of the ko00860 pathway (porphyrin and chlorophyll metabolism) and the increased abundance of *Cutibacterium* [81,82]. Although this KEGG pathway includes chlorophyll metabolism, only the porphyrin-related processes are biologically relevant in this context. Porphyrins generate complement component C5 chemotactic factors, which act as cofactors in initiating pro-inflammatory stress. The degradation of C5 can lead to the generation of C5a, triggering an inflammatory response [83]. Excessive inflammation can cause autoimmune disorders, allergic reactions, and other inflammatory conditions, including alopecia [84].

Increased fatty acid biosynthesis (ko00061) was observed in the AGA group. Short-chain fatty acids (SCFAs), such as propionate and valerate produced by *C. acnes*, can inhibit histone deacetylase (HDAC) activity in keratinocytes and promote cytokine expression in response to Toll-like receptor (TLR) ligands, including TLR2 and TLR3 [85]. These outcomes can lead to epithelial inflammation. Similarly to porphyrin, the synthesis of short-chain fatty acids (SCFAs) such as propionate and valerate also likely increased. Therefore, future studies on hair loss should investigate the reasons behind the increase in *Cutibacterium* due to changes in the hair follicle layer environment and how its metabolites affect hair follicle cells, sebaceous glands, stem cells, and other related structures. *Corynebacterium*, which produces antibacterial free fatty acids from triacylglycerols, inhibits bacteria such as *Streptococcus pneumoniae* and enhances human skin stability against external factors [86]. This reduction may be linked to increased *Cutibacterium* levels, potentially limiting the production of *Corynebacterium*-derived free fatty acids which contribute to skin barrier defense.

Certain microorganisms can utilize human steroid hormones [87]. The decrease in steroid hormone biosynthesis (ko00140) observed in the AGA group is likely due to microbial imbalance, reducing the levels of microorganisms that traditionally utilize existing steroid hormones. This imbalance may also have led to the excessive accumulation of steroid hormones. In AGA, testosterone is converted into dihydrotestosterone (DHT) by five alpha-reductases. DHT binds to androgen receptors in hair follicle cells, reducing the production of growth factors that stimulate hair matrix cell proliferation or inducing factors that inhibit hair growth, thereby suppressing hair growth [88]. In women, even when testosterone is secreted, the pattern of AGA can differ. This difference is due to the higher conversion of testosterone into the female hormone estradiol, particularly in the frontal region where aromatase is more abundant [89]. Steroid hormones such as testosterone and estradiol directly impact immune system regulation, bacterial metabolism, growth, and the expression of toxic factors [90,91]. Due to distinct steroid hormone profiles in females and males, it is crucial to investigate how specific microbial groups utilize steroid hormones. Moreover, studying changes in metabolites resulting from steroid hormone treatments on the microbiome and understanding how these metabolites affect human cells will be essential for advancing treatments for both female and male pattern hair loss.

This study has several limitations. First, we did not perform the direct quantification of steroid hormone levels due to sample type constraints. Instead, we relied on PICRUSt2-based functional inference, which does not capture gene expression or metabolite levels. Second, 16S rRNA V3–V4 sequencing limits the taxonomic resolution, especially at the species level. Third, the absence of a healthy male control group restricts direct sex-based comparisons in follicular microbiota. Nevertheless, our analysis offers new data on the microbiome of hair follicle layers in AGA patients and provides hypotheses for future investigation.

## 5. Conclusions

Building on these findings, our study presents the first comprehensive analysis of the hair follicle layer microbiome in female and male AGA patients, revealing distinct alterations in microbial diversity, composition, and predicted metabolic functions. These findings underscore the potential role of follicular microbes in the pathophysiology of AGA, particularly in FPHL. By identifying key microbial taxa and functional pathways, our work establishes a foundation for future translational studies and highlights new opportunities for microbiome-based diagnostics and therapies in hair loss.

To advance beyond predictive inference, future studies should incorporate targeted functional assays to validate how microbial metabolites—such as porphyrins, short-chain fatty acids, or steroid intermediates—interact with specific cell types within the hair follicle, including dermal papilla cells, germinal matrix keratinocytes, and perifollicular immune cells. For instance, in vitro coculture systems or ex vivo follicle organoids may provide insight into how these microbial products influence cytokine signaling, hormonal metabolism, and epithelial differentiation. Moreover, integrated metatranscriptomic or metabolomic profiling, in combination with hormonal quantification, would allow for the more precise elucidation of microbe–host interactions. These approaches will be essential to determining whether the observed taxonomic and functional shifts are causally implicated in the pathophysiology of AGA, particularly in sex-specific contexts.

## Figures and Tables

**Table 1:** The LDA scores and relative abundance of microbiota biomarkers in the AGA groups. Significant taxa were identified using LEfSe analysis in the FPHL, MPHL, and control groups. The top six taxa with the highest discriminative power (LDA ≥ 2.0) are shown for each group. The relative abundance values represent the mean proportion of each taxon within each group, calculated from within-sample proportions based on ASV-level taxonomic assignments. The taxonomic classification followed the SILVA 138v database.

Group	Genus and Species	LDA Score	Case.F	Case.M	Control
FPHL	*Phyllobacterium*	3.466	0.488	0.000	0.000
	*Selenomonas*	3.153	0.038	0.000	0.010
	*Streptococcus*	2.882	0.173	0.047	0.168
	*Acinetobacter*	2.771	0.038	0.018	0.022
	*Caulobacter*	2.708	0.057	0.019	0.033
	*Veillonella*	2.658	0.086	0.001	0.038
MPHL	*Cutibacterium*	5.299	62.579	78.954	42.135
	*Pseudomonas*	4.490	0.143	5.810	4.072
	*Cutibacterium granulosum*	3.528	0.152	0.659	0.030
	*Oscillospira* uncultured bacterium	3.113	0.000	0.000	0.007
Control	*Lawsonella*	5.060	16.398	3.542	27.096
	*Lawsonella* uncultured bacterium	5.060	16.394	3.542	27.091
	*Bacteroides*	4.110	0.172	0.093	2.446
	*Clostridium* *septicum*	3.894	0.005	0.000	1.519
	*Clostridium sensu stricto 1*	3.882	0.145	0.212	1.606
	*Bacteroides* *caccae*	3.865	0.020	0.007	1.372

**Table 2:** The different abundance of six genera in AGA and other scalp disease patients. The proportions of specific genera within the hair follicle layer or scalp microbiome analysis results have been delineated for patients experiencing hair loss conditions such as androgenetic alopecia and alopecia areata, as well as for those with scalp disorders including atopic dermatitis, scalp pruritus, dandruff, and seborrheic dermatitis.

Disease	Androgenetic Alopecia					Alopecia Areata		Atopic Dermatitis	Scalp Pruritus	Dandruff	Seborrheic Dermatitis
Skin area	Hair follicle layers		Scalp								
Sex	Female	Male	Female	Male	Male	Male	Female, Male				Male
Country	Korea				Japan	Korea	Italia	Korea	China		
Author	Our study		Jung et al., 2022 [15]		Suzuki et al., 2021 [16]	Won et al., 2022 [58]	Pinto et al., 2019 [57]	Woo et al., 2022 [55]	Li et al., 2022 [56]	Grimshaw et al., 2019 [54]	Lin et al., 2021 [67]
*Cutibacterium*	↑	↑	↓	↓	↑	ns	↑	↓	↓	↓	ns
*Lawsonella*	↓	↓	↓		-	-	-	↓	ns	-	-
*Staphylococcus*	↑	↓	↓	↓	↑	↓	↓	↑	↓	↑	↑
*Pseudomonas*	↓	↑	↓	↑	↓	-	-	-	↓	-	↓
*Corynebacterium*	↓	↑	↓	↑	↓	↑	-	↑	↑	-	-
*Micrococcus*	↓	↓	↓	↑	↓	-	-	-	-	-	-

↑: Case > control; ↓: case < control; ns: not significant; -: not described.

## Data Availability

The information on metagenome datasets generated during the current study is included in this article, and it is available from the corresponding authors upon reasonable request.

## Supplementary Materials

The following supporting information can be downloaded at: https://www.mdpi.com/article/10.3390/microorganisms13061365/s1, Table S1: Clinical sample information. Table S2: Bacterial ASV taxonomy classification. Table S3: Alpha-diversity of hair follicle layer microbiome. Table S4: Alpha-diversity statistical analysis results. Table S5: Unweighted UniFrac and Bray–Curtis distance analysis results. Table S6: Comparison of relative abundance in FPHL, MPHL, and control groups. Table S7: LEfSe analysis results through LDA scores and *p*-values. Table S8: PICRUSt2 functional analysis feature list and *p*-values.
